# Open-Coast Eelgrass (*Zostera marina)* Transplant Catalyzes Rapid Mirroring of Structure and Function of Extant Eelgrasses

**DOI:** 10.1007/s12237-025-01609-x

**Published:** 2025-09-30

**Authors:** Rilee D. Sanders, Adam K. Obaza, David W. Ginsburg, Olivia C. Carmack, Benjamin C. Grime, Heather Burdick, Tom K. Ford, James J. Leichter

**Affiliations:** 1 Scripps Institution of Oceanography, University of California San Diego, La Jolla, CA USA; 2 Paua Marine Research Group, Long Beach, CA USA; 3 Environmental Studies Program, University of Southern California, Los Angeles, CA USA; 4 The Nature Conservancy, Los Angeles, CA USA; 5 The Bay Foundation, Los Angeles, CA USA

**Keywords:** Restoration, Seagrass, *Zostera marina*, Ecosystem recovery, Ecosystem services, Biodiversity

## Abstract

Seagrasses are marine angiosperms that function as ecosystem engineers, forming complex structure that enhance nearshore environments. Globally, seagrass habitats are threatened by intensifying impacts from climate change, which exacerbate non-climatic stressors such as coastal development, invasive species, and overfishing. Advances in the methodological efficacy of active seagrass restoration efforts have sought to mitigate substantial anthropogenic-induced losses. Restoration efforts along the U.S. West Coast have primarily focused on *Zostera marina* (common eelgrass) in shallow, sheltered estuarine environments, where most coastal development occurs. However, within the Southern California Bight, *Zostera* spp. also occurs along the exposed coastlines of the California Channel Islands archipelago. Despite their unique location and the ecosystem services they provide, a paucity of information persists on open-coast seagrass systems and restoration efforts. In this study, we conducted a novel transplant of *Z. marina* on Catalina Island and tracked temporal and spatial performance metrics (i.e., areal coverage, morphometrics, and fish assemblages) at the restoration site and seven extant *Z. marina* reference beds on the island from 2021 to 2024. The transplant activities successfully established over 0.18 hectares of *Z. marina* habitat. The transplant site paralleled or exceeded extant reference beds morphometrically (shoot density and blade length) and functionally (fish composition and fish diversity), while concomitantly providing habitat for the occupancy of, and utilization by, federally listed endangered and managed species. Our results provide a model for broadening the scope of, and augmenting strategies for, seagrass habitat recovery beyond conventional restoration spaces by underscoring the role of open-coast seagrasses in enhancing nearshore ecosystem function and resilience.

**Supplementary Information:**

The online version contains supplementary material available at 10.1007/s12237-025-01609-x.

## Introduction

The precipitous decline of biodiversity, and the loss of associated habitat functions, in the nearshore coastal environment catalyzed by anthropogenic activities is stark (Halpern et al., 2008; Jackson et al., 2001; Talukder et al., 2022). Accordingly, coastal foundational species are recognized as a high priority by regulatory agencies at the local, state, regional, and federal levels (Lotze et al., 2006; NOAA, 2014). Growing efforts to protect and recover foundational species in accordance with regulatory guidance have led to the development of restoration programs (Bayraktarov et al., 2016; Danovaro et al., 2021; Duarte et al., 2020; Eger et al., 2022). Yet restoration activities are inherently challenging (Valdez et al., 2020), and despite considerable methodological advancements, failure rates remain high (van Katwijk et al., 2016). Beyond the task of active species recovery, practitioners must define defensible success metrics (Ward & Beheshti, 2023). A project’s outcome is often best defined by utilizing readily detectable structural attributes (e.g., density and areal coverage) (Suding et al., 2011) in concert with metrics that describe habitat services and functions (e.g., fish nursery function and carbon storage) (Beheshti et al., 2022). The structural recovery of a foundational species, taken in amalgam with the recovery of habitat function, allows for direct evaluation of project success and highlights core motivations for undertaking restoration efforts (Orth et al., 2020).

Seagrasses are a group of marine monocotyledonous angiosperms distributed in nearshore systems that form critical foundational habitat structure and function (Boström et al., 2006; Duarte, 2002; Unsworth & Cullen-Unsworth, 2014). Seagrasses function as ecosystem engineers, creating complex biogenic structure in systems otherwise limited by three-dimensional habitat (Duffy, 2006). Habitat structured by seagrasses provides multiple ecosystem services supporting diverse fish and invertebrate assemblages (Heck et al., 2003; Hughes et al., 2009; Irlandi et al., 1999; Tanner et al., 2019) and bolstering the capacity to support extractive fisheries (Nordlund et al., 2018; Unsworth et al., 2019b). In some cases, seagrasses can act as “blue carbon” systems through the fixation and storage of biological carbon (Duarte & Krause-Jensen, 2017; Duarte et al., 2013; Ward et al., 2021) which can partially ameliorate the local impacts of ocean acidification (Kapsenberg & Hofmann, 2016; Ricart et al., 2021; Unsworth et al., 2012). Providing provisioning services, buffering coastal erosion (Hansen & Reidenbach, 2012), or reducing harmful bacteria in the water column (Reusch et al., 2021), it is evident that seagrasses contribute substantially to improved quality of life for humans (Herrera et al., 2022; McKenzie et al., 2021). Despite the ecological, economic, and cultural value of seagrasses (Barbier et al., 2011), seagrass habitats are jointly exposed to acute anthropogenic impacts (e.g., shoreline development, eutrophication) (Short & Wyllie-Escheverria, 1996; Holon et al., 2015; Eriander, 2017) and accentuated by the accelerating disturbances from climate change (e.g., sea surface temperature fluctuations, sea level rise) (Jung et al., 2023; Turschwell et al., 2021; Unsworth et al., 2022). Extensive declines in the extent, health, and stability of seagrass habitats evince their vulnerability to a myriad of ongoing stressors (Krause-Jensen et al., 2020) and erode their capacity to deliver critical ecosystem services and functions.

Seagrass habitats along the North-Eastern Pacific are dominated by *Zostera marina* (common eelgrass) primarily inhabiting estuaries and bays (Blok et al., 2018; Munsch et al., 2023; Short et al., 2007). *Zostera* spp. habitats, especially in California, encounter substantial anthropogenic impacts (Kelly et al., 2019) that can alter key biological (Altstatt et al., 2014; Hughes et al., 2013) and biophysical (Magel et al., 2023; Serrano et al., 2021; Skelton et al., 2024; Wong et al., 2021) parameters driving seagrass stability and ultimately catalyze severe losses (O’Leary et al., 2021). As an avenue to conserve, protect, and enhance these critical foundational habitats, regulatory agencies have implemented “no net loss” policies (Levrel et al., 2012) and require compensatory mitigation when direct impacts (e.g., dredging, dock construction) to *Zostera* spp. result in habitat degradation (Bernstein et al., 2011; NOAA, 2014). On-site and in-kind mitigation and restoration are preferred as an avenue to directly offset losses (Pausch et al., 2024), and efforts have been primarily constrained to estuaries, bays, and harbors (Ward & Beheshti, 2023). A significant challenge arises as the region experiences a geographical constriction of suitable habitat for estuarine seagrass restoration driven by climatic alterations (e.g., sea level rise, thermal stress) (DuBois et al., 2022; Echavarria-Heras et al., 2006; Johnson et al., 2003) and anthropogenic stressors (e.g., eutrophication, coastal development) (Obaza et al., 2015; Kelly et al., 2019; O'Leary et al., 2021). This habitat compression, coupled with the documented 75% decline in California’s estuarine vegetation over the past century (Stein et al., 2020), underscores the need to diversify restoration and enhancement strategies to buttress against further losses.

The Southern California Bight (SCB) encompasses ~ 1200 km of coastline along the mainland and the California Channel Islands, an eight-island archipelago consisting of highly diverse and economically valuable subtidal marine ecotones (Fautin et al., 2010; Miller, 2023; Williams et al., 2022). A direct function of the regional oceanographic dynamics, anthropogenic activities (e.g., sedimentation, urban pollution, eutrophication) (North, 1963; Schiff & Bay, 2003), and biophysical gradients (e.g., sea surface temperature, pH) (Gelpi, 2023; Williams et al., 2022) manifest stronger impacts along the mainland coastline compared to the offshore islands. Catalina Island experiences less frequent and smaller magnitude disturbance than the mainland and more northwesterly located islands, which, in conjunction with the relative isolation from deleterious anthropogenic impacts, underpins the ecological patterns observed (Claisse et al., 2018). However, Catalina Island, the only island in the archipelago with a permanent civilian population of ~ 4200 and with over one million annual visitors, is not completely protected from anthropogenic induced impacts. The island’s robust tourism industry and rich biodiversity represent a critical resource for a multidimensional stakeholder assemblage including indigenous tribes, recreational and commercial fishing, tourists, and scientists (Iacchei et al., 2005; Looby & Ginsburg, 2021; Pondella & Allen, 2000). Regulatory regimes managed to curb historical over-exploitation on the island (Collier, 2020; Parnell et al., 2006; Pondella, 2009), and the island remains a popular destination (Tompkins & Steller, 2016) with productive and biodiverse ecosystems (Ginsburg & Huang, 2022; Tanner et al., 2019). Because anthropogenic conditions continue to be more adverse for coastal habitats along the mainland, there is tremendous value in restoration of less impacted areas (i.e., an offshore island) unbounded by space and with complementary high chance of success.

As *Z. marina* habitats are primarily associated with shallow, protected estuarine environments, a lack of information exists on the remote and oft-isolated critical open-coast seagrass habitats. This data gap challenges global inventories of seagrass coverage (McKenzie et al., 2020). For example, spatial distributional data for the Mediterranean seagrass *Posidonia oceanica* (Telesca et al., 2015; Traganos et al., 2022), deep-water seagrass along Australia’s Great Barrier Reef region (Coles et al., 2009; York et al., 2015), and broadscale seagrass coverage in South Australia (Clarke et al., 2021) remain poorly documented. Although estuarine seagrass restoration has achieved notable success (Beheshti et al., 2022; Gräfnings et al., 2023; Orth et al., 2020), open-coast efforts are primarily constrained to Australia (Paling et al., 2001, 2003, 2007; Tan et al., 2020) and a few other examples (Paulo et al., 2019; Wegoro et al., 2022). This open-coast seagrass data paradigm extends to the SCB and Catalina Island specifically, where kelp forest research is abundant (Bushing, 1996; Ginsburg & Huang, 2022; House & Allen, 2022; Klingbeil et al., 2022; Pondella et al., 2005; Zahn et al., 2016; Zimmerman & Robertson, 1985), but open-coast seagrass studies are scarce (Obaza et al., 2022). Notable exceptions include open-coast *Zostera* spp. distributional and morphometric surveys in the Channel Islands archipelago (Engle & Miller, 2005), the elucidation of a genetic gradient among *Z. marina* and *Zostera pacifica* (wide-leaved eelgrass) (Watson, 1890) across the SCB (Coyer et al., 2008; Olsen et al., 2014), and investigations of *Zostera* spp. fish habitat function and fisheries dynamics (Obaza et al., 2022; Tanner et al., 2019). Sanders et al. (2024) conducted the first mainland open-coast *Z. pacifica* transplant in the SCB, demonstrating that while open-coast *Zostera* spp. persist beyond estuarine-based biophysical envelopes, transplant survival remains strongly driven by exceedances of biophysical thresholds and by transplant methodology, underscoring the critical role of informed site selection in the efficacy of open-coast restoration. In advance of this present study, recent collaborative survey efforts determined *Z. marina* beds located on the eastern leeward side of Catalina Island to be reduced or missing altogether, despite no salient changes in habitat suitability (Obaza et al., 2022; Obaza, unpublished data), impelling the need for restorative actions.

The loss of seagrass on Catalina Island represents a unique opportunity to build off burgeoning open-coast temperate seagrass research and develop transplant methodology for open-coast *Z. marina* beds. The purpose of this study was to utilize best available science from prior successful *Zostera* spp. transplant efforts and implement the methodologies in a novel setting (i.e., Catalina Island) to create open-coast *Zostera* spp. restoration techniques and concomitantly determine the efficacy of enhancing open-coast temperate seagrass ecosystem services and habitat function. Specifically, the aim of our study was to (1) track the spatial and temporal survivorship of transplanted *Z. marina* on Catalina Island and (2) assess restoration success through a determination of the mechanistic structural attributes that crosswalk to restored ecosystem function. The utilization of discrete deployments of biophysical sensor arrays during the site selection process allowed for quantifiable confirmation of transplant site suitability. Quarterly monitoring of spatial and temporal *Z. marina* performance and fish assemblages at the transplant site, in concert with extensive distributional, morphometric, and fish assemblage monitoring of seven reference sites, occurred from 2021 to 2024. This monitoring regime provided the framework for substantiating the capacity to effectively restore and enhance open-coast *Z. marina* habitat. These efforts progress the nascent understanding of temperate open-coast seagrass habitat and greatly augment the ability of resource managers, academics, and restoration practitioners to move beyond conventional restoration spaces and expand the scope of habitat restoration and improved ecosystem function towards oft ignored, yet high-valued habitats (i.e., open-coast).

## Materials and Methods

### Study Area

**Fig. 1:**
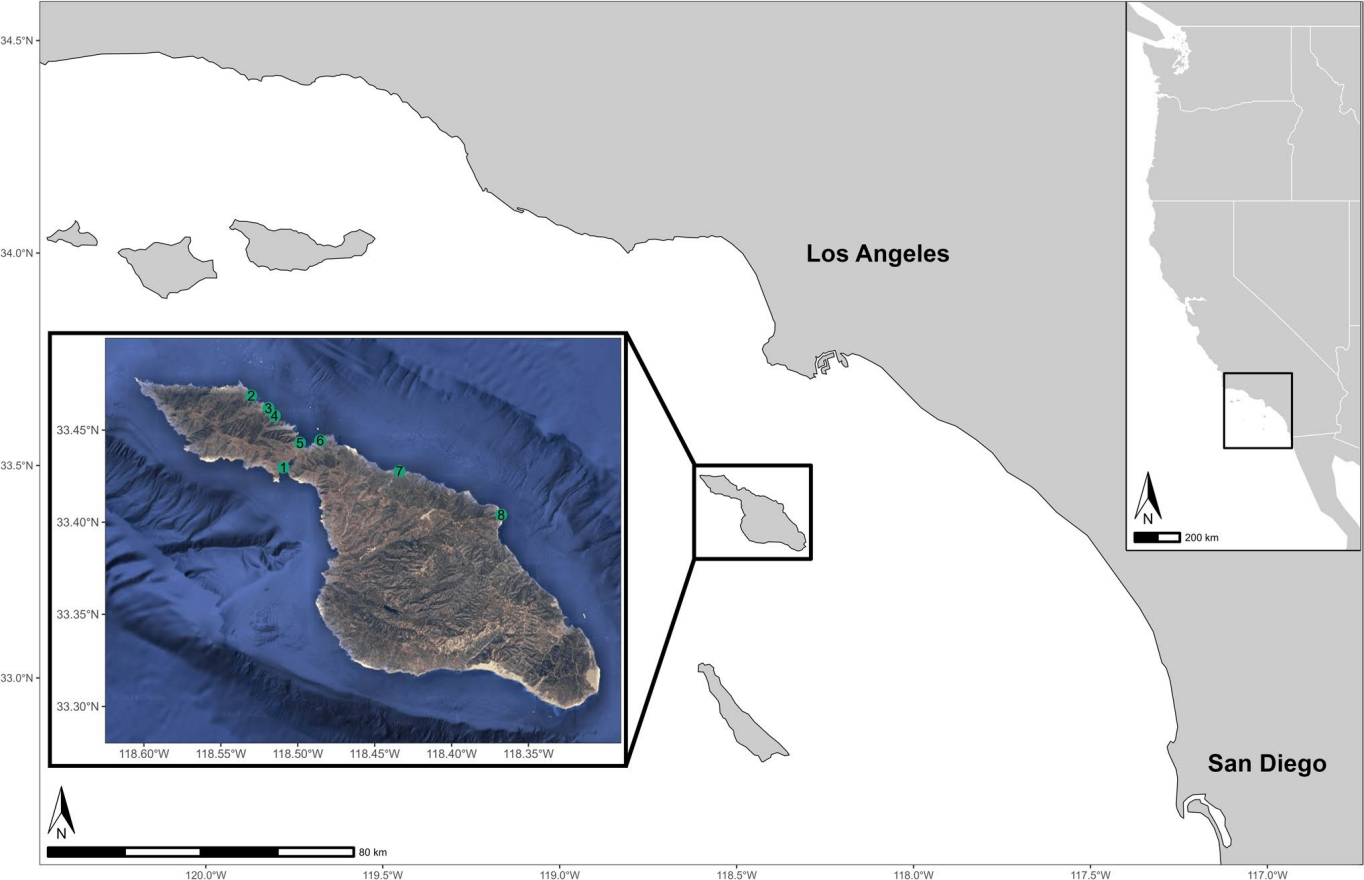
Locations of *Zostera marina* (common eelgrass) transplant site and reference bed sites on Catalina Island, with respect to the Southern California Bight. Starting on the windward side of the island and moving clockwise, sites (indicated by number) included (1) Catalina Harbor, (2) Emerald Bay, (3) Big Geiger Cove, (4) Little Geiger Cove, (5) Two Harbors, (6) Big Fisherman’s Cove, (7) Ripper’s Cove, and (8) Button Shell

The angle of the coastline of the Southern California Bight (SCB) begets a convergence of subarctic waters from the north and subtropical waters from the south (Bray et al., 1999; Hickey, 1992), marking a biological transition zone between the Oregonian marine biogeographic province to the north and the Californian province to the south (Claisse et al., 2018; Hamilton et al., 2010; Pondella et al., 2005), explicating the degree of subtidal habitat productivity and biodiversity. Positioned in a southeasterly locale within the SCB, Catalina Island, the third largest island (196 km^2^) in the archipelago, was the location of the study, with the project focused along the leeward (northern) coastline (Fig. 1). The *Zostera marina* (common eelgrass) transplant site was established at Button Shell (33.4039, −118.3677), which formerly contained a robust bed (Engle & Miller, 2005), but which has been absent since at least the onset of monitoring by Obaza et al. (2022) in 2018. The transplant site area was delineated in 6–11 m depth, typical of natal open-coast *Z. marina* beds within the SCB (Obaza et al., 2022). Donor material was harvested from Ripper’s Cove (33.4277, −118.4337), historically the largest *Z. marina* bed on Catalina Island (Obaza et al., 2022) and located 7 km from Button Shell. Six additional reference *Z. marina* beds were located primarily along the Leeward side of the island at distances ranging from 13 to 38 km from the transplant site.

### Biophysical Monitoring

In the SCB, open-coast seagrass transplanting is in a nascent stage and necessitates biophysical sampling efforts to characterize the environment (Sanders et al., 2024). At the onset of the study (June–July 2022; *n* = 28 days), we measured continuous in situ benthic photosynthetically active radiation (PAR), dissolved oxygen (DO), and temperature at the donor and transplant sites to assess site suitability based on general similarity of biophysical conditions. Sensor arrays were deployed at 7.6 m depth at both the transplant and donor sites.

PAR was measured continuously as photosynthetic photon flux density (PPFD) using miniPAR (Precision Measurement Engineering) optical time-series instruments sampling at a 10-min interval. The PPFD data (µmol m^−2^ s^−1^) were integrated over the course of each sampling day, creating daily integrated PAR (mol quantum m^−2^ day^−1^) following Dunic and Côté (2023). DO was measured using a submersible miniDOT (Precision Measurement Engineering) instrument which detects oxygen concentrations in the water column sampling at a 10-min interval. Benthic temperature was measured with a thermistor instrument (both miniDOT and miniPAR, Precision Measurement Engineering) sampling at a 10-min interval.

### Donor Material Collection

The *Z. marina* donor material was harvested from Ripper’s Cove, a robust extant bed on the Leeward side of Catalina Island, covering 0.59 hectares with a shoot density of 306 shoots per m^2^ at depths of 5–12 m. Although collecting donor material from multiple geographically distinct sites can enhance genetic diversity and reduce harvest impacts (Williams & Davis, 1996; NOAA, 2014; Olsen et al., 2014), and while numerous other extant *Z. marina* beds exist along the leeward side of Catalina Island (Engle & Miller, 2005; Obaza et al., 2022), permit limitations precluded collection from those sites. To likely increase clonal genetic diversity, divers harvested shoots from shallow, middle, and deep sections of the donor bed following Olsen et al. (2014).

Donor material collection followed the methodological approach described in Sanders et al. (2024). Material for both the single and bundle shoot methodologies was harvested using a thinning approach to minimize disturbance. Shoots were gently extracted with at least three internodal rhizome segments (~ 100 mm) intact (Altstatt et al., 2014), without actively removing sediment (Paling et al., 2007). Donor material collected for the single shoot transplant method was stored in shaded, flow-through seawater coolers aboard the vessel and transported directly to the transplant site within 1 h. Donor material for the bundle shoot transplant method was transported (~ 20 min) to the USC Wrigley Marine Science Center, held in shaded flow-through water tables, bundled with biodegradable twine following Zhou et al. (2014), and then returned to coolers for transport (~ 60 min) to the transplant site.

### Transplant

In June 2022, aligning with the peak growing season (March–October) defined by NOAA’s 2014 California Eelgrass Mitigation Policy (CEMP), *Z. marina* donor material was harvested from Ripper’s Cove and transplanted the same day to Button Shell to minimize stress. At the transplant site, SCUBA divers established a 120 m baseline with seven 21 m perpendicular transects, creating six replicate plots (420 m^2^ each) along the same depth strata gradient. A random number generator was utilized to determine which transplanting methodology (single shoot or bundle shoot) would occur in each transplant plot. Transplant plots zero, one, and five received the single shoot method, transplant plots three and four received the bundle shoot method, and transplant plot two was left fallow (Fig. S1 in Supplementary Materials). The transplant approach followed Sanders et al. (2024). Material was planted in a grid pattern within each plot, and meter sticks were used to maintain transplant spacing. For the bundle shoot transplant method, 350 bundles (consisting of 3500 shoots) were planted at 1 m intervals within both transplant plots three and four. In bundle shoot plots, divers placed the entire bundle into an excavated hole, securing a wooden tongue depressor to the rhizome mass with biodegradable twine and positioning it parallel to the substrate to serve as an anchor before backfilling the hole with sediment. For the single shoot transplant method, 650, 100, and 650 shoots were planted at 0.5 m intervals within transplant plots zero, one, and five, respectively. In single shoot plots, divers carefully maneuvered a single rhizome into the sediment, securing the rhizome with a small gardening stake (Altstatt et al., 2014). Across both transplanting methods and all transplanting plots, a total of 8400 shoots were collected and cumulatively transplanted at Button Shell, constituting the first open-coast *Z. marina* transplant and only transplant on Catalina Island reported to date.

### Biological Monitoring

Fish community and eelgrass morphometric surveys were conducted at the donor bed prior to and after collection of transplant material in accordance with permitting requirements. Post-transplant monitoring of the transplant site, Button Shell, occurred 1 month post-transplanting and at quarterly intervals thereafter through July 2024. Annual growing season surveys were conducted at the seven reference *Z. marina* beds (including the donor bed) on the Leeward side of Catalina Island from 2021 to 2024. While the frequency of reference site monitoring may limit detection of high-temporal-resolution trends (i.e., intra-annual), annual monitoring is sufficient to capture inter-annual variability (Malone et al., 2022; Obaza et al., 2022; Pondella et al., 2019; Williams et al., 2021), striking a critical balance between scientific defensibility and logistical or financial constraints, particularly given the parallel investment in active restoration.

Timed roving diver fish community surveys were conducted at each site. Each survey was completed in 1.5–5 min in which divers swam < 1 m above the substrate and identified the species, enumerated the abundance, and determined the size of each fish within a 1 m high × 2 m wide survey (per Looby & Ginsburg, 2021; Obaza et al., 2022; Pondella et al., 2006). Collection of morphometrics (length, width, and density) of *Z. marina* followed methods in Obaza et al. (2022). Blade length, blade width, and shoot density were measured in situ within 0.07 m^2^ quadrats, haphazardly placed at > 1 m intervals throughout the vegetated *Z. marina* habitat (n ≥ 30). Density was recorded as the total number of shoots per quadrat and scaled to calculate shoots per m^2^, while the blade length and width measurements correspond to a representative shoot within each quadrat. The areal coverage of *Z. marina* reference and transplant sites was quantified using methods described in Obaza et al. (2022) which utilized a GPS receiver (Trimble® R1), enabled with a Satellite-Based Augmentation System, and interfaced with a smartphone to provide real-time sub-meter accuracy during mapping operations. These surveys were accomplished via SCUBA.

### Time Lapse Camera Monitoring

To assess species utilization of the transplant site at higher resolution time-intervals and with less disturbance than achievable with divers, a time lapse camera (TLC) was deployed at Button Shell. The TLC was comprised of a HD-SDI zoom camera (SubAqua Imaging Systems) with a 3-W strobe configuration set to take a photo at 30-min intervals and was affixed < 1 m from the substrate in the middle of the transplant site. Four separate TLC deployments occurred across 12 separate months from June to July 2022, February to March 2023, May and August 2023, and January to April 2024. Following the conclusion of deployments, TLCs were recovered by divers and images downloaded for further processing and analysis.

### Statistical Analysis

#### Biophysical Metrics

Generalized Linear Models (GLM) were used to assess differences in seagrass biophysical regimes (Tuya et al., 2019; de los Santos et al., 2020; Twomey et al., 2023; Kindeberg et al., 2024) between transplant and donor bed sites. A log–log-link Gamma GLM was fit to account for the continuous, strictly positive, and slightly right-skewed data (Quinn & Keough, 2002; Crawley, 2015; Zurr et al., 2009). The biophysical metric (PAR, DO, or temperature) was the response variable, with site (transplant or donor) as a two-level categorical predictor. Models were fitted in R using the “glm” function from the “stats” package, and model assumptions were assessed via visual inspection of residual plots and diagnostic tools from the “performance” package (Lüdecke et al., 2021). GLM diagnostics indicated acceptable fits for all three biophysical variables, with no influential outliers (Cook’s distance < 0.5), residual uniformity (*p* ≥ 0.105), and dispersion ratios near 1 (PAR: 0.872; DO: 0.948; temperature: 1.024). Empirical cumulative distribution function (eCDF) plots were employed to differentiate patterns of the environmental metrics among sites (Beheshti et al., 2022). Standardization of the time series date range was applied to environmental data for statistical analysis (Sanders et al., 2024). All analyses and visualizations were completed in R (R Core Team, 2023, v. 4.3.2).

#### Biological Data

Structural morphometrics (i.e., seagrass density and blade length) were examined for normality utilizing the Shapiro–Wilk test (Razali & Wah, 2011) from the “stats” package in R (R Core Team, 2023, v. 4.3.2) along with Q-Q plots, while homogeneity of variance was tested using Levene’s test (Bennett et al., 2022). As the transplant site did not exist in 2021 and was only partially established in 2022, morphometric data for statistical analysis was constrained from 2023 to 2024, in order to prevent unbalanced testing (Shaw & Mitchell-Olds, 1993). Shoot density and blade length data for individual years (2023 and 2024) were non-normally distributed (Shapiro–Wilk *p* < 0.05) and analyzed using log-link GLMs (Crawley, 2015; Quinn & Keough, 2002; Zuur et al., 2009). Site (eight-level categorical) was included as a fixed effect in both models, which were fitted in R using the “glm” function from the “stats” package. Model assumptions were assessed via visual inspection of residual plots and diagnostic tools from the “performance” package (Lüdecke et al., 2021). GLM diagnostics indicated satisfactory fits, with no influential outliers (Cook’s distance < 0.5), non-significant residual uniformity, and no evidence of dispersion issues. Combined-year data also failed normality tests (Shapiro–Wilk *p* < 0.05), so analysis was extended to log-link Gamma Generalized Linear Mixed Models (GLMMs) to account for the multi-year, multi-site structure (Zuur et al., 2009), common in seagrass ecological and restoration studies (Costa et al., 2021; Gagnon et al., 2021; Graham et al., 2024; Hardison et al., 2023; Orfanidis et al., 2021; Watson et al., 2023; Yang et al., 2016). GLMMs were fitted in R using the “glmTMB” package (Brooks et al., 2017) with site as a random effect and included transplant status, year, and their interaction as fixed effects. Including site significantly improved model fits for both shoot density (ΔAIC = 205, *χ*^2^(1) = 206.87, *p* < 0.001) and blade length (ΔAIC = 130, *χ*^2^(1) = 131.83, *p* < 0.001), indicating substantial site-level variation. Pairwise comparisons were conducted using the “emmeans” package with a Tukey adjustment (Lenth, 2024). Model validation, including residual simulations (*n* = 5,000) via the “simulateResiduals” function from the “DHARMa” package (Hartig, 2024), confirmed satisfactory fits for shoot density and blade length, with non-significant residual uniformity (*p* ≥ 0.176), appropriate dispersion (shoot density: 0.764; blade length: 0.851), and variance inflation factors below multicollinearity thresholds (shoot density: VIF < 2.26; blade length: VIF < 1.97).

Differences in the composition of fish assemblages among reference and transplant sites were compared by utilizing the “vegdist” function in the “vegan” package in R (Oksanen et al., 2019). All fish count data were relativized to create species-specific percent composition values per transect, which were then averaged to produce relative species-specific abundance data per sampling event at a specific survey site. A Bray–Curtis dissimilarity matrix was created from this mean relative species-specific abundance data for the sites from 2021 to 2024. Two-dimensional, non-metric multi-dimensional scaling (NMDS) plots were used to visualize patterns and changes in fish assemblages across transplant and reference sites over time using the “metaMDS” function in the “vegan” package. We performed a Durbin-Watson test to assess potential autocorrelation of NMDS scores among study years (Shuster et al., 2022), with neither NMDS axis 1 (*p* = 0.956) nor NMDS axis 2 (*p* = 0.496) having a significant result, indicating a lack of temporal serial correlation. To test for spatial autocorrelation, we constructed a geographical distance matrix using the “distHaversine” function from the “geosphere” package (Hijmans, 2024). We then conducted Mantel tests (Mantel, 1967) using the “mantel” function from the “vegan” package, testing the mean relative species-specific abundance Bray–Curtis dissimilarity matrix and the geographical data distance matrix (Legendre, 1993; Legendre & Legendre, 2012; Van Mantgem & Schwilk, 2009). The results of the Mantel test (*p* = 0.901) indicated that spatial autocorrelation was not detected in the data. Differences in fish assemblage (permutational multivariate analysis of variance, PERMANOVA; McCune & Grace, 2002) were determined using the “adonis” function in the “vegan” package. Since PERMANOVA tests are sensitive to dispersion (Anderson, 2001), an analysis of multivariate homogeneity (PERMDISP) tested the differences in group dispersion using the “betadisper” function in the “vegan” package, confirming non-significant dispersion prior to initiating any PERMANOVA tests.

To determine species-specific differences in fish assemblage, an indicator species analysis (ISA; Dufrêne & Legendre, 1997) was performed with transplant status (i.e., transplant vs reference site) as a factor utilizing the “multipatt” function from the “indicspecies” package (De Cáceres & Legendre, 2009). The “diversity” function in the “vegan” package was used to calculate alpha diversity metrics (i.e., species richness and Shannon–Wiener Diversity Index) commonly used in seagrass ecological studies (Deudero et al., 2008; Duffy et al., 2003; Hughes & Stachowicz, 2009). Alpha diversity metric data were normally distributed (Shapiro–Wilk *p* = 0.277 and 0.701 for species richness and Shannon Diversity Index, respectively), and ANOVA tests were conducted to assess differences in fish assemblages across transplant status, sites, and sample year.

#### Time Lapse Camera Data

A Microsoft Access database was constructed for the processing and analysis of the Time Lapse Camera (TLC) images (see Obaza et al., 2023 for further details). A form was filled with records of fish species presence in each image that subsequently populated tables within the same database. Once all TLC images were processed, they were exported to R (R Core Team, 2023, v. 4.3.2) for data cleaning and statistical analysis.

To address fish species as the transplant site progressed from initial planting, fish species were identified to the lowest taxonomical level and enumerated in each image. Species-specific counts were summed across day, month, and seasonal timeframes. Fish species data were further classified into individual feeding guilds following methodology reported in Obaza et al. (2022). Guilds were determined by foraging mode and may be found in Appendix A of Bond et al. (1999). The “diversity” function in the “vegan” package was used to calculate measures of alpha diversity (i.e., species richness and Shannon–Wiener Diversity Index). A Durbin-Watson test was used to assess potential autocorrelation of alpha diversity metrics across month and seasonality with neither species richness (*p* = 0.232 and 0.321) nor Shannon–Wiener Diversity Index (*p* = 0.364 and 0.287) having a significant result, indicating a lack of temporal serial correlation. Alpha diversity metrics from the TLC data were normally distributed (Shapiro–Wilk *p* = 0.860 and 0.393 for species richness and Shannon Diversity Index, respectively), and ANOVA tests were conducted to assess differences in fish abundance across months and seasons. Linear regressions were conducted, where alpha diversity metrics were the response variable and seasonality the predictor variable.

## Results

### Biophysical Metrics

**Fig. 2:**
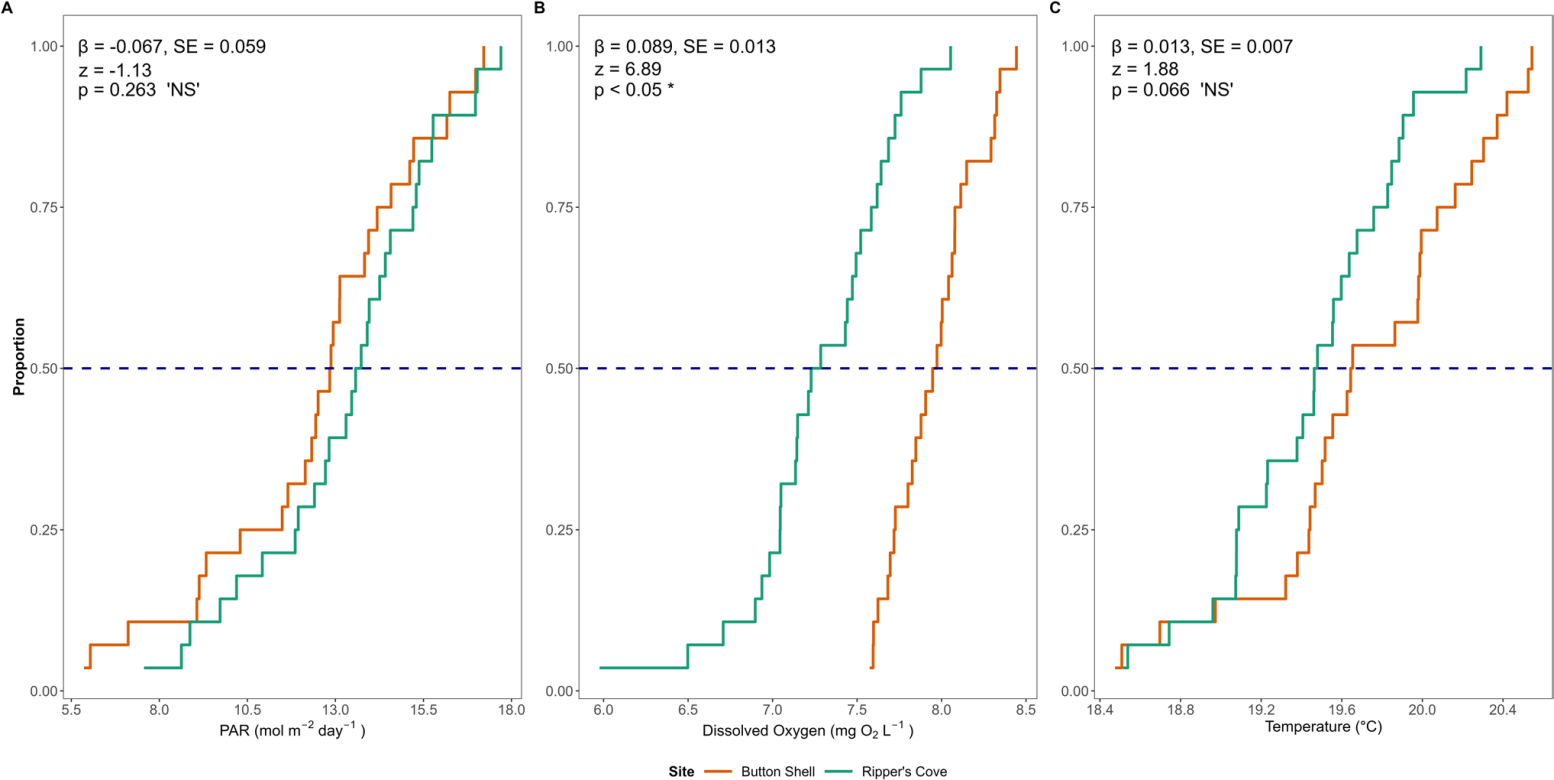
Empirical cumulative distribution function (eCDF) curves for **A** photosynthetically active radiation (mol m^−2^ day^−1^), **B** dissolved oxygen (mg O_2_ L^−1^), and **C** temperature (°C) at transplant (Button Shell) and donor bed (Ripper’s Cove) sites. Plotted PAR values are daily integrated data, while DO and temperature values are mean daily data. The intersection between eCDF and the horizontal blue dashed line represents the median (50%) value at each site. Log-link Gamma Generalized Linear Model (GLM) results are reported on plots

The biophysical sensor arrays at both Button Shell (transplant site) and Ripper’s Cove (donor site) were deployed from June to July 2022 (*n* = 28 days). Photosynthetically active radiation (PAR) sensors (*n* = 4032 measurements per site) recorded an average daily mean value of 12.42 mol m^−2^ day^−1^ at the transplant site and 13.28 mol m^−2^ day^−1^ at the donor site (Fig. 2A). The lowest daily mean PAR value recorded at the transplant site was 5.86 mol m^−2^ day^−1^ while the lowest value at the donor bed was 7.56 mol m^−2^ day^−1^. While the GLM results estimated that PAR at the transplant site was 6.49% lower compared to the donor bed, the PAR regimes at transplant and donor bed sites were not significantly different (*β* = −0.067, SE = 0.059, *z* = −1.13, *p* = 0.263).

Similarly, sensor arrays at transplant and donor sites recorded dissolved oxygen (DO) profiles (*n* = 4032 measurements per site), with an average daily mean value of 7.95 mg O_2_ L^−1^ at the transplant site and 7.27 mg O_2_ L^−1^ at the donor site (Fig. 2B). The lowest daily mean DO value recorded at the transplant site was 7.57 mg O_2_ L^−1^ while the lowest value at the donor bed was 5.98 mg O_2_ L^−1^. The GLM indicated that DO at the transplant site was 9.35% higher compared to the donor bed and that the DO regimes at transplant and donor bed sites were significantly different (GLM *β* = 0.089, SE = 0.013, *z* = 6.89, *p* < 0.05), yet noting that neither site experienced values below critical tissue degradation threshold values (3 mg O_2_ L^−1^ identified by Moore and Jarvis, 2008).

Sensor arrays at transplant and donor sites recorded temperature regimes (*n* = 8064 measurements per site), with an average daily mean value of 19.44 °C at the donor site and 19.70 °C at the transplant site (Fig. 2C). The highest daily mean temperature value recorded at the transplant site was 20.5 °C, while the highest value at the donor site was 20.3 °C. While the GLM estimates that temperature at the transplant site was 1.33% higher compared to the donor bed, the temperature regimes at transplant and donor bed sites were not significantly different (*β* = 0.013, SE = 0.007, *z* = 1.88, *p* = 0.066).

### Transplant Expansion

**Fig. 3:**
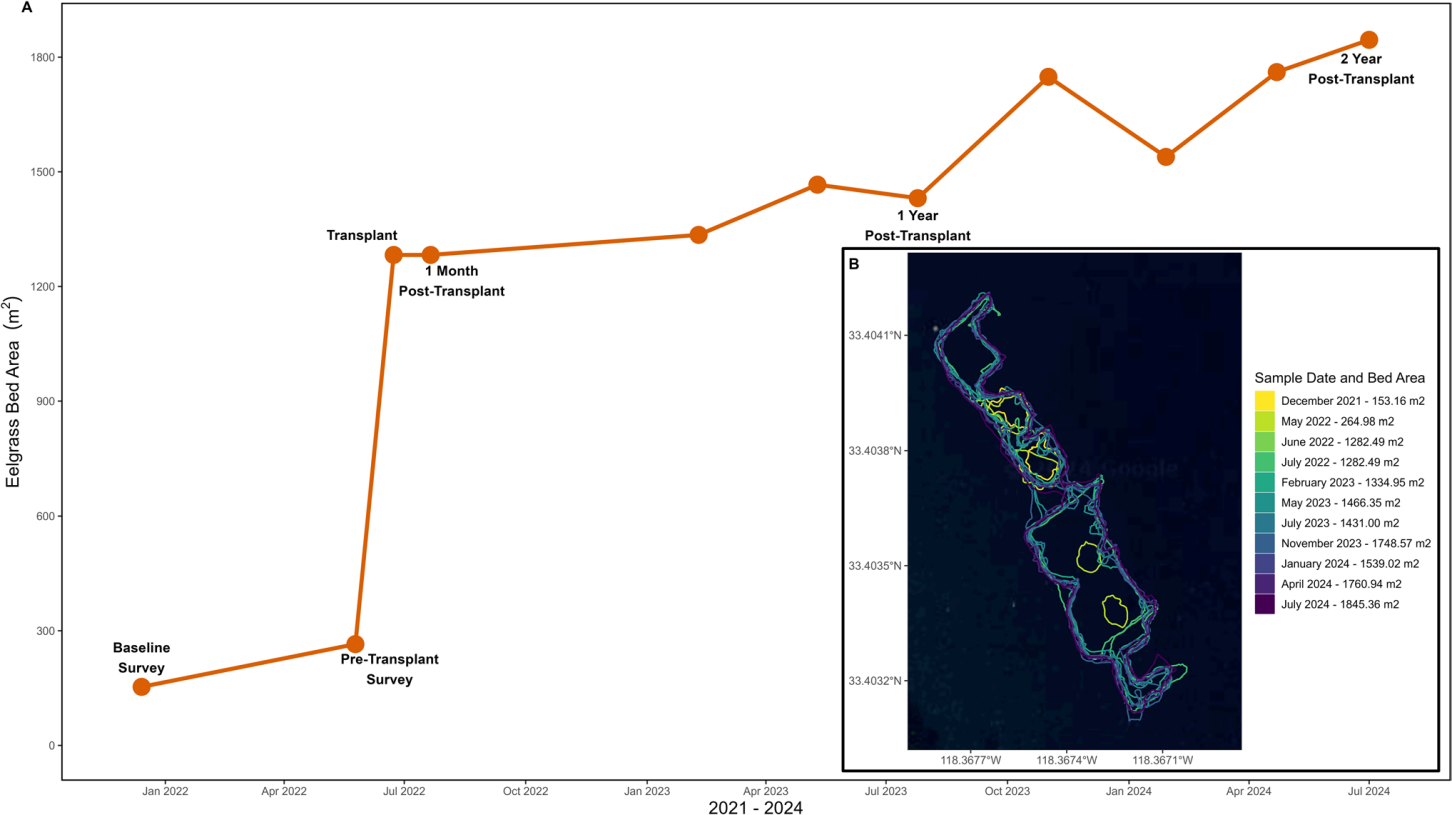
*Zostera marina* (common eelgrass) bed area (m.^2^) at the Button Shell transplant site from December 2021 through July 2024 visualized **A** graphically and **B** via aerial map of transplant extent. Transplanting activities occurred in June 2022

The Button Shell transplant site retained the transplanted material without suffering a substantial initial reduction (at the 1-month survey) in transplanted area, before steadily gaining ~ 150 m^2^ in transplant area by the 1-year post-transplant survey, and ~ 563 m^2^ by the 2-year post-transplant survey (Fig. 3A). The transplanting effort effectively created a net total of over 1580 m^2^ of new habitat with an overall size of 0.18 hectares at Button Shell (Fig. 3B). Across the study period, there were only two sampling events that detected reductions in area, a 35.3 m^2^ reduction between May and July 2023, and a 209 m^2^ reduction between November 2023 and January 2024, noting however that each reduction was followed by increases exceeding the bed area prior to the reduction.

Uneven performance across the transplant plots (1-month post-transplanting survivorship ranged from 47 to 99% and 8-month post-transplanting survivorship ranged from 35 to 463%) still resulted in net expansion of the transplant area. Across the 8-month post-transplanting period (June 2022–February 2023), average survivorship by transplant method was 116% for single shoot and 51% for bundle shoot. From February 2023 onwards, the Button Shell site experienced growth resulting in transplanted *Z. marina* filling in spaces between transplant units (i.e., individual single shoot or bundle shoot units) and expanded outside of respective plot boundaries, making further inter-transplanting plot tracking unfeasible.

### Eelgrass Morphometrics

#### Density

As the transplant site did not exist in 2021 (density = 0 shoots per m^2^) and was established in June of 2022 (with a site density of 5.08 ± 1.47 SE shoots per m^2^), morphometric data for statistical analysis was constrained to 2023 to 2024 (1-year and 2-year post-transplanting).

Button Shell ranked 7 out of 8 of the sites surveyed for density in 2023 (*n* = 487) and mimicked the shoot density at low-end reference sites, with no significant difference in blade length detected between the transplant site and Catalina Harbor (rank 8) nor Big Fisherman’s Cove (rank 6) (*p* = 0.087 and 0.144 respectively). In 2024 (*n* = 470), Button Shell density was consistent with reference beds with medium densities relative to the others, ranking 4 out of 8 total sites, with no significant differences in shoot density detected between the transplant site and Ripper’s Cove (rank 5) (*p* = 0.103).

**Fig. 4:**
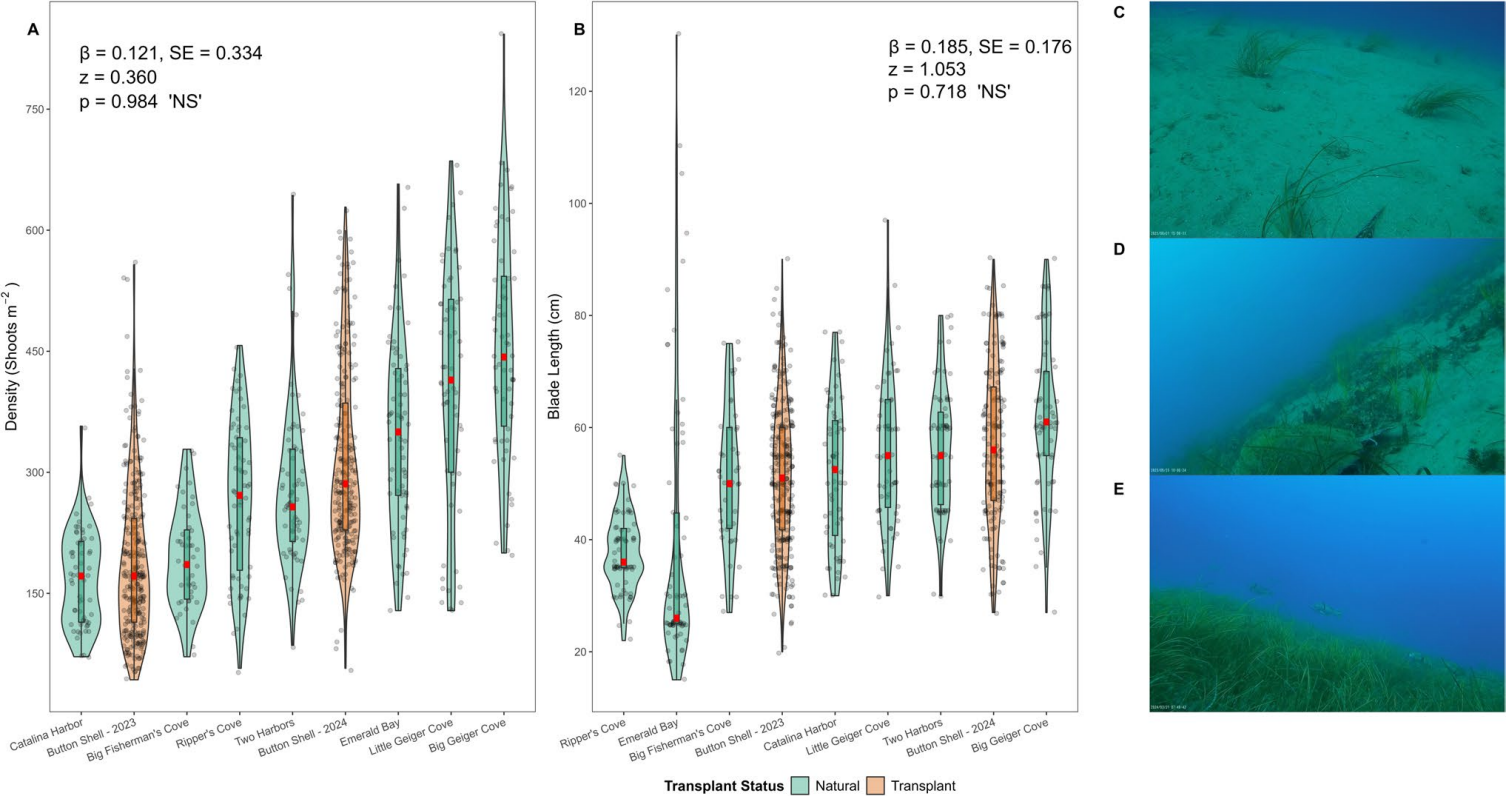
Violin plots, with internal box plots, of eelgrass morphometrics: **A** density and **B** blade length at the transplant and reference bed sites from 2023 to 2024. Color represents transplant status. Red dot represents median (50%) value at each site. Log-link Gamma Generalized Linear Model (GLM) test results to assess the differences between transplant and reference sites are reported on plots. Images from the bottom mounted Time Lapse Camera at Button Shell visually represent changes in morphometrics as the transplant site progresses: **C** June 2022 shows dispersed bundle shoot transplanting units, **D** May 2023 shows patchy but expanded eelgrass occupied by U.S. Endangered Species Act listed *Chelonia mydas* (Green Sea Turtle), and **E** March 2024 shows densely established and robust eelgrass bed

Combining the 2 years of post-transplanting data (2023 to 2024, *n* = 957), the mean shoot density at the transplant site (*n* = 528) was 244 shoots per m^2^ ± 5.52 SE and 306 shoots per m^2^ ± 6.95 SE across reference beds (*n* = 429). GLMM results indicate that in 2023, shoot density was not significantly lower in the transplant site relative to reference sites (*β* = −0.463, SE = 0.334, *z* = −1.38, *p* = 0.166). A significant effect of year was detected, with density in reference sites decreasing in 2024 relative to 2023 (*β* = −0.077, SE = 0.039, *z* = −1.97, *p* < 0.05). The shoot density at the transplant site increased from 2023 to 2024 (*β* = 0.584, SE = 0.053, *z* = 11.12, *p* < 0.001; Fig. 4A). Pairwise comparisons of estimated marginal means (EMMs) supported these patterns, as shoot density at the transplant site in 2024 did not significantly differ from shoot density at reference sites in either 2023 (*β* = 0.044, SE = 0.335, *z* = 0.13, *p* = 0.999) or in 2024 (*β* = 0.121, SE = 0.334, *z* = 0.36, *p* = 0.984).

#### Blade Length

Consistent with the density data, blade length data was also constrained to 1-year and 2-year post-transplanting (2023 to 2024) for data analysis.

Button Shell ranked 6 out of 8 of the sites surveyed for blade length in 2023 (*n* = 469), with no significant difference in blade length detected between the transplant site and Catalina Harbor (rank 6), Little Geiger Cove (rank 5), nor Two Harbors (rank 4) (GLM *p* = 0.093, *p* = 0.913, *p* = 0.463, respectively). In 2024 (*n* = 392), the blade length at Button Shell exceeded low-end reference beds and mimicked mid and high-end reference beds, ranking 4 out of 8 total survey sites for mean blade length, with no significant difference detected between the transplant site and Catalina Harbor (rank 5), Two Harbors (rank 3), nor Little Geiger (rank 2) (GLM *p* = 0.919, 0.598, and 0.236, respectively).

Combining the 2 years of post-transplanting blade length data (*n* = 861), the mean blade length at the transplant site (*n* = 432) was 53.4 cm ± 0.657 SE and 50.1 cm ± 0.818 SE across reference sites (*n* = 429). GLMM results indicate no significant differences in blade length between transplant and reference sites in 2023 (*β* = −0.024, SE = 0.175, *z* = −0.14, *p* = 0.889). Blade lengths at reference sites decreased in 2024 relative to 2023 (*β* = −0.104, SE = 0.027, *z* = −3.86, *p* < 0.001), indicative of a significant year effect. Conversely, an increase in blade length at the transplant site from 2023 to 2024 was evident (*β* = 0.209, SE = 0.039, *z* = 5.42, *p* < 0.001; Fig. 4B). Pairwise comparisons of EMMs confirmed these results, as blade lengths at the transplant site in 2024 did not significantly differ from blade lengths at reference sites in either 2023 (*β* = 0.081, SE = 0.176, *z* = 0.459, *p* = 0.968) or in 2024 (*β* = 0.185, SE = 0.176, *z* = 1.053, *p* = 0.718).

### Fish Surveys

#### Fish Community

Scientific divers completed 200 roving visual fish surveys in which they spent 969 min (16.2 h) underwater across eight survey sites from 2021 to 2024. During these surveys, 16,760 individual fishes from 33 different species were recorded. Nine species (*Atherinopsis affinis* (2.12%), *Cymatogaster aggregata* (6.86%), *Halichoeres semicinctus* (31.90%), *Oxyjulis california *(3.51%), *Paralabrax clathratus* (20.85%), *Sardinops sagax* (2.09%), *Trachurus symmetricus* (14.70%), *Umbrina roncador* (2.64%), and *Xenistius californiensis* (12.25%)) accounted for > 96% of fishes encountered, with four species (*Halichoeres semicinctus* (31.90%), *Paralabrax clathratus* (20.85%), *Trachurus symmetricus* (14.70%), and *Xenistius californiensis* (12.25%)) comprising ~ 80% of fish observations.

**Fig. 5:**
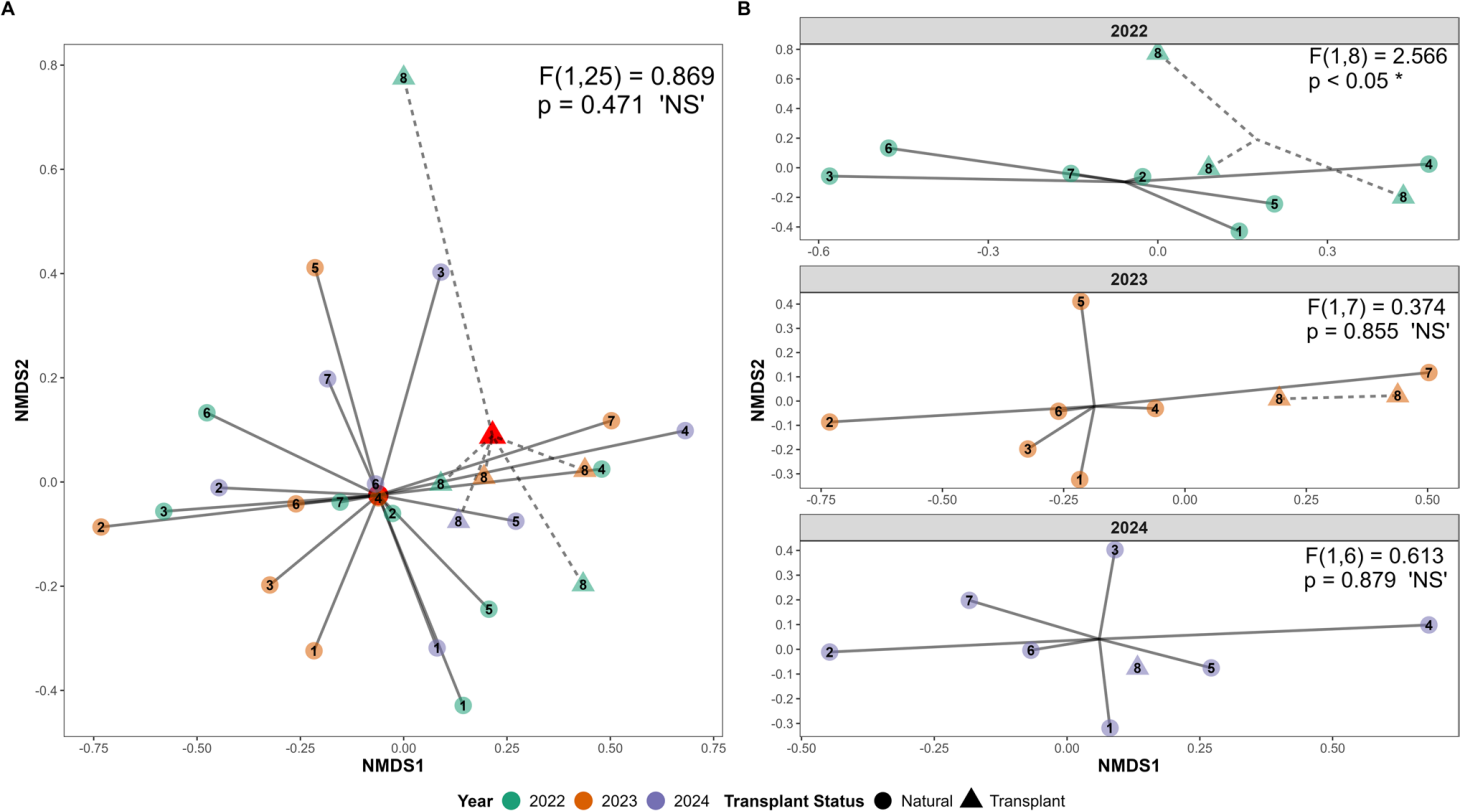
Non-metric multidimensional scaling (NMDS) plots depict the Bray–Curtis dissimilarity matrix, where each point is the mean fish assemblage from each site by sampling event (stress = 0.14) for **A** all data and **B** on a per-year breakdown. Color represents survey year, shape represents transplant status, and the number within each shape corresponds to a specific site (see Fig. 1 for site names). Red shape represents transplant status specific calculated centroid. Permutation multivariate analysis of variance (PERMANOVA) results to assess differences between transplant and reference sites are reported on plots. NMDS shows that community composition is initially dispersed and significantly different (in 2022) but rapidly mirrors reference beds with no significant difference detected in 2023 and 2024

PERMANOVA results for 2022 to 2024 indicated that fish assemblage composition at transplant and reference bed sites did not differ significantly overall (*F*_1,25_ = 0.869, *p* = 0.471; Fig. 5A). However, fish assemblage composition varied significantly among sites (*F*_7,19_ = 2.087, *p* < 0.01), underscoring localized spatial variation. Visualization of fish assemblage by transplant status across individual years (Fig. 5B) revealed an initial significant difference in 2022 (*F*_1,8_ = 2.566, *p* < 0.05), which disappeared in 2023 (*F*_1,7_ = 0.374, *p* = 0.855), and remained nonsignificant in 2024 (*F*_1,6_ = 0.613, *p* = 0.879). An analysis of multivariate homogeneity of group dispersions (PERMDISP) indicated that including data from 2021 to 2024 resulted in significant dispersion differences (*F*_1,32_ = 6.238, *p* = 0.018), likely driven by the 2021 data in which the transplant site was not yet established and thus lacked grouping structure. To avoid unbalanced testing (Anderson & Walsh, 2013), the fish assemblage PERMANOVA was constrained from 2022 to 2024, during which group dispersion was not significantly different (*F*_1,25_ = 4.208, *p* = 0.051), supporting more reliable interpretation of detected group differences.

Indicator Species Analysis (ISA) showed the nine most observed fish species listed above (i.e., *Halichoeres semicinctus*, *Paralabrax clathratus*, etc.) did not significantly differ between transplant and reference beds. Of the 33 species observed and tested, only two species (*Pleuronichthys* spp. and *Heterodontus francisci)* were significantly associated with the transplant site. Though of note, this is likely primarily driven by infrequent sightings of a relatively small number of observations: three total observations of *Pleuronichthys* spp. (two at the transplant site and one at a reference bed) (0.02% of total fish observed) and six observations of *Heterodontus francisci* (all at the transplant site) (0.04% of total fish observed).

#### Alpha Diversity

**Fig. 6:**
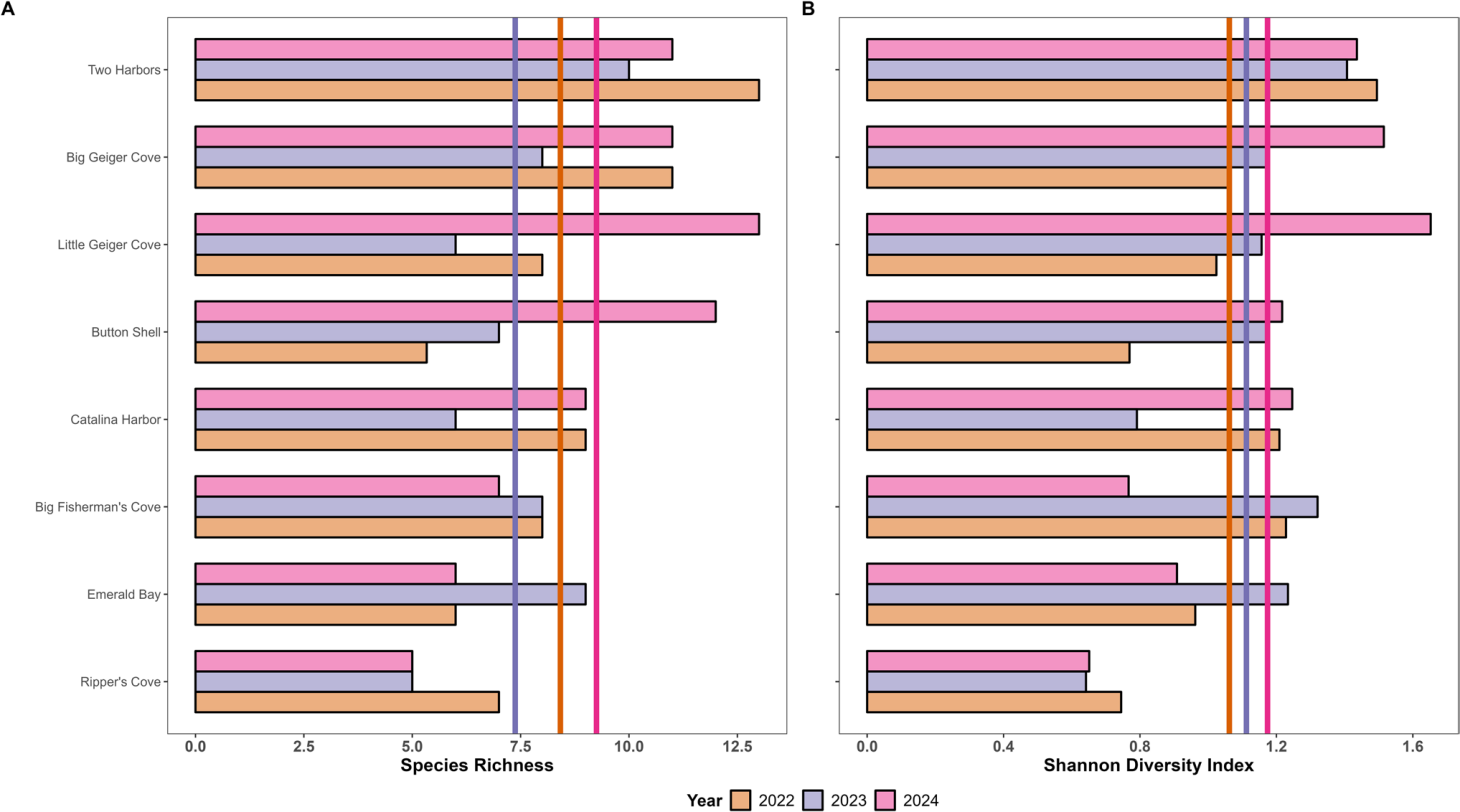
Alpha diversity metrics, **A** Species Richness and **B** Shannon Diversity Index, calculated based on the fish abundance data for each site from 2022 to 2024. Color represents year. Vertical lines indicate a yearly average across all sites

The mean species richness from 2022 to 2024 (normal distribution) was 8.38 at reference sites and 8.11 at the transplant site and was not significantly different (ANOVA *F*_1,25_ = 1.382, *p* = 0.251) (Fig. 6A). The Two Harbors site had the highest species richness (11.3), followed by Big Geiger Cove and Little Geiger Cove (10.0 and 9.0 respectively), and then Button Shell (8.1) ranking 4 of 8 survey sites, and noting that species richness did not significantly differ by site (ANOVA *F*_7,19_ = 2.088, *p* = 0.096) nor by year (ANOVA *F*_2,24_ = 1.313, *p* = 0.288), with 2024 having the highest species richness value.

The mean Shannon Diversity Index from 2022 to 2024 (normal distribution) was 1.125 at reference sites and 1.052 at the transplant site and was not significantly different (ANOVA *F*_1,25_ = 1.183, *p* = 0.287) (Fig. 6B). The Two Harbors site had the highest Shannon Diversity Index value (1.446), and Ripper’s Cove had the lowest value (0.679), noting Shannon Diversity Index significantly differed by site (ANOVA *F*_7,19_ = 2.864, *p* < 0.05), but did not significantly differ by year (ANOVA *F*_2,24_ = 0.786, *p* = 0.467), with 2024 having the highest Shannon Diversity Index value.

### Time Lapse Camera Data

A total of 10,330 photos were captured across 12 separate deployment months, of which 17.35% (1792 images) had a fish observation recorded. Species observations combined to 14,474 from 29 distinct species. Species were matched to 13 separate foraging guilds where six guilds: (1) schooling pelagic foragers (*Scomber japonicus*, *Trachurus symmetricus*, *Atherinops affinis*, and *Atherinopsis californiensis*), (2) crushers (*Halichoeres semicinctus*, *Bodianus pulcher*, and *Rhacochilus vacca*), (3) non-schooling, diurnal, engulfers (*Paralabrax clathratus* and *Heterostichus rostratus*), (4) herbivores (*Medialuna californiensis* and *Girella nigricans*), (5) benthic foragers, schooling, diurnal, pickers (*Oxyjulis californica*, *Brachyistius frenatus*, and *Cymatogaster aggregata*), and (6) nocturnal, generalists (*Umbrina roncador* and *Anisotremus davidsonii*), accounted for over 98% of total species observed in the images. Schooling pelagic foragers accounted for 84% of observations alone. The most common elasmobranch species observed were *Myliobatis californica*, *Rhinobatos productus*, and *Urobatis halleri*. Of particular note were the repetitive (3) separate images which captured the visitation of the U.S. Endangered Species Act listed *Chelonia mydas* (Green Sea Turtle) at the transplant site (Fig. 4D), as well as by federally managed rockfish species (*Sebastes paucispinis*).

**Fig. 7:**
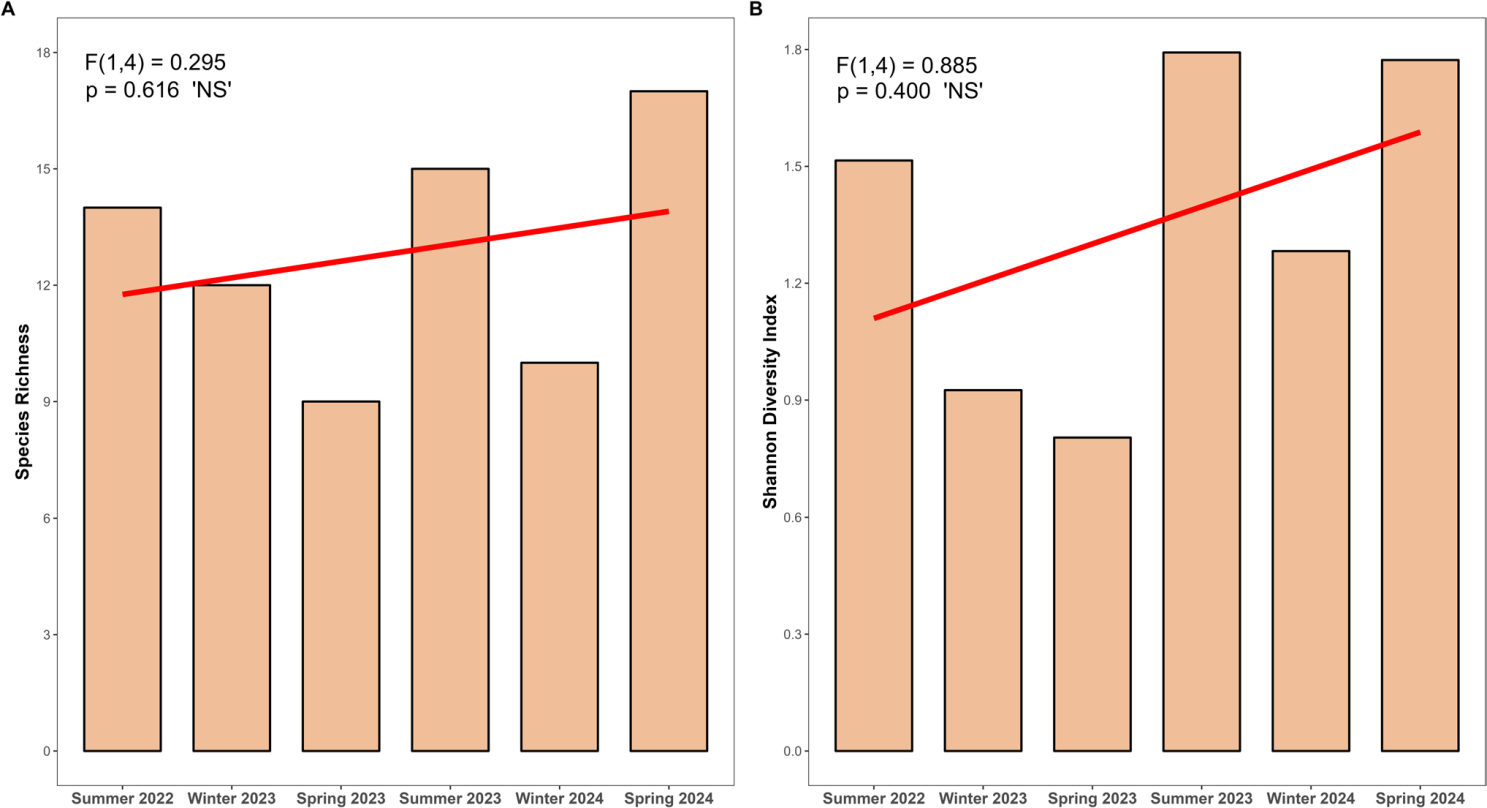
Alpha diversity metrics, **A** Species Richness and **B** Shannon Diversity Index, calculated from Time Lapse Camera (TLC) fish abundance data by season. Analysis of variance (ANOVA) test results to assess differences in alpha diversity metrics across seasons are reported on plots. Linear regression lines, where alpha diversity metrics were the response variable and seasonality the predictor variable, are displayed on plots

The mean species richness at the transplant site across the six seasonal timeframes (normal distribution) was 12.83. No significant difference was detected by season (ANOVA *F*_1,4_ = 0.295, *p* = 0.616), though Spring 2024 had the highest species richness (17) and the GLM had a positive trajectory as time post-transplanting progressed (Fig. 7A). Summer exhibited the highest species richness (14.5), followed by Spring (13) and Winter (11). It is also noted that species richness did not significantly differ across individual months either (ANOVA *F*_1,10_ = 0.080, *p* = 0.783).

The mean Shannon Diversity Index at the transplant site across the six seasonal timeframes (normal distribution) was 1.35 and did not significantly differ by season (ANOVA *F*_1,4_ = 0.885, *p* = 0.400). The GLM had a positive trajectory as time post-transplanting progressed (Fig. 7B). Summer also displayed the highest Shannon Diversity Index (1.65), followed by spring (1.28) and winter (1.10). It is also noted that Shannon Diversity Index did not significantly differ across individual months either (ANOVA *F*_1,10_ = 0.861, *p* = 0.375).

## Discussion

The restoration activities conducted at Button Shell represent the first holistic offshore island *Zostera marina* (common eelgrass) transplanting effort, which successfully created 0.18 hectares of novel eelgrass habitat that morphometrically and functionally mirrored or exceeded extant reference beds on the island within 2 years. Taken in concert with the rapid utilization of restored area by a diverse fish assemblage, including federally managed and ESA listed species, these results are indicative of successful restoration and enhancement of nearshore habitat. While success is often defined solely in terms of the target species (in this case, the foundational *Z. marina*) (Suding et al., 2011), our study goes beyond survivorship to offer a holistic evaluation of success, focusing on habitat function as indicated by the presence of a broad community of associated fauna (Ward & Beheshti, 2023).

### Structural Recovery

Site selection is one of the most critical steps in seagrass restoration (Rehlmeyer et al., 2024; Short et al., 2002). The transplant site at Button Shell formerly contained a stable, sizable, and robust bed (Engle & Miller, 2005) but was absent since at least 2018 (Obaza et al., 2022). Bed loss was associated with impacts from a rare, intense hurricane that caused severe coastal damage and degraded water quality (Los Angeles Times, 2014; J. Selbitschka pers. Comm., 15 February 2025). While not previously reported for the Southern California Bight, storm-driven deleterious impacts to other seagrass systems are well documented (Feehan et al., 2024; Pérez-Estrada et al., 2021; Short & Wyllie-Echeverria, 1996; Yue et al., 2021). This loss was a stochastic disturbance rather than a chronic degradation, and thus, no long-term changes in site suitability were observed (e.g., no landslides or shading from new permanent overwater structures)–confirmed through baseline surveys. Although admittedly deployed over a short timeframe, in situ biophysical sensor arrays provide quantitative confirmation of site suitability (Fig. 2), with light, dissolved oxygen, and temperature remaining within growth-supportive ranges for seagrasses (Dunic & Côté, 2023; Lee et al., 2007; Moore & Jarvis, 2008; Thom et al., 2008; Vaquer-Sunyer & Duarte, 2008), consistent with conditions at other open-coast *Zostera* sites on Catalina Island (Sanders et al., 2024). Prior to transplanting, we confirmed the absence of the causative agent for *Z. marina* loss at Button Shell as per van Katwijk et al. (2016). These results suggest that sites where unvegetated habitat stemmed from acute, rather than chronic, disturbance may remain viable for restoration, provided press biophysical stressors are absent.

After transplanting at Button Shell, the seagrass bed expanded to 0.18 hectares (Fig. 3), aligning with pre-collapse estimates (0.1–1 ha; Engle & Miller, 2005). The transplant maintained its area for the first month, a period typically marked by severe reductions in seagrass restoration projects (Rezek et al., 2019), and likely facilitated by the large initial transplant size (Gräfnings et al., 2023). The transplant continued expanding with minor seasonal fluctuations, though sustained establishment remains uncertain due to limited knowledge of open-coast *Zostera* spp. drivers, including disease susceptibility (Yang et al., 2023), climate stress (Sanders et al., 2024), and site-specific variations (Obaza et al., 2022). Even so, Button Shell showed strong survivorship when contextualized to other regional open-coast *Zostera* spp. projects, which experienced 70–95% mortality within 6 months (Altstatt et al., 2014; Sanders et al., 2024). While a full methodological survivorship analysis is absent from this study, the twofold higher initial survival of the single shoot transplants underscores the importance of transplant technique (Fonseca et al., 1998). Variation in methodological survivorship may reflect impacts from localized bat rays (*Myliobatis californica*) foraging behavior (Meese & Lowe, 2019; Fernández-Aguirre et al., 2022; Grew et al., 2025), as observed by divers and time-lapse cameras in our study or by the likely increased drag on larger-profiled bundle shoots (Orth et al., 1999), particularly in wave-exposed, open-coast environments (Sanders et al., 2024). By year one, structural attributes (i.e., shoot density and blade length) emulated reference beds, and in some cases, surpassed reference beds by year two (Fig. 4), with rhizomatic extension facilitating rapid colonization of adjacent bare sediments (Fonseca et al., 2004; Jensen & Bell, 2001). Global restoration sites typically reach community structures similar to natural beds within 2 to 3 years (Beheshti et al., 2022; Sievers et al., 2025), although full equivalence may take up to 6 years (Bell et al., 2014), underscoring the rapid structural recovery at Button Shell.

Numerous studies from other regions (Orth et al., 2020; Tassone et al., 2024) document rapid structural recovery, including Gagnon et al. (2023), which reported a twofold increase in eelgrass biomass and shoot density in transplanted plots within 1 year. In our study, transplanting donor material over numerous plots (van Katwijk et al., 2009), distributed over a relatively large site area (Gräfnings et al., 2023), aligns with the notion of spreading risk (Suykerbuyk et al., 2016). Taken in concert with the rapid achievement of densities similar to reference beds in our study likely satisfied the population size threshold (van Katwijk et al., 2016) obligated to catalyze density-dependent positive feedback at the transplant site (Allee, 1931; Paulo et al., 2019; Rehlmeyer et al., 2024; Valdez et al., 2020). Transplant sizes in excess of critical thresholds allow for self-facilitation processes. Specifically, transplanted seagrass enhances water clarity by reducing bioturbation and preventing sediment erosion through rhizome stabilization (Carr et al., 2012; de Boer, 2007; Suykerbuyk et al., 2016), potentially accelerating site expansion. In contrast, a prior open-coast transplant failed driven by adverse biophysical conditions (Sanders et al., 2024). Although structural mirroring of transplanted seagrass to natural beds is not a direct requirement to support a similar community (Tanner et al., 2021), structural recovery of foundational species often facilitates functional recovery (Beheshti et al., 2022; Dayton, 1972).

### Functional Recovery

Habitat restoration remains extremely challenging, in part because a multitude of underlying mechanisms and feedback loops remain poorly understood (Nordlund et al., 2024). This issue is compounded by the propensity for failed projects to go unreported (Unsworth et al., 2023), contributing to low success rates (van Katwijk et al., 2016). Habitat restoration actions are generally promoted as leading to the recovery of ecological function (O’Brien et al., 2022), but this relationship may not be straightforward (Ehrenfeld, 2000). Indeed, the guiding seagrass policy document in California does not necessitate assessing functional recovery, rather utilizing seagrass distribution and density as proxies for function (NOAA, 2014). This approach may be practical given that mitigation project resources are often insufficient to support conventional levels of scientific rigor. However, the underlying principle persists: although attention to habitat structure is valuable, the functional capacity of the habitat holds greater ecological relevance and interest to stakeholders. Therefore, evaluating the recovery of ecosystem function remains essential for a more accurate assessment of restoration success (Tanner et al., 2021). As such, the opportunity to take the next step in linking restored seagrass habitat function (Castro-Fernández et al., 2025; Hori et al., 2009; McSkimming et al., 2016; Orth et al., 2020) to novel habitat (i.e., open-coast) is both timely and unique. The present study is the first extensive inquiry of open-coast *Z. marina* transplanting, incorporating morphometric and functional habitat metrics to enable a comprehensive evaluation of functional recovery.

This study, conducted from 2021 to 2024 across seven reference beds and one transplant site, demonstrated that the Button Shell transplant increased fish abundance, richness, and diversity and that the fish assemblage of the transplant site rapidly mirrored reference sites. Benchmarking transplant site recovery against reference sites provides for a comprehensive evaluation of successful habitat function reestablishment (Gräfnings et al., 2024). Within 1 year, the fish assemblage at the transplant site rapidly converged with reference sites, indicating rapid ecological stabilization. While no significant differences were observed between transplant and reference sites in 2023 and 2024, limited intra-annual replication warrants caution in interpreting direct ecological equivalence. Nonetheless, annual monitoring remains a standard approach in ecological field sampling and is specifically effective at detecting community variability in open-coast seagrass studies (Obaza et al., 2022). The transition from unvegetated to vegetated habitat at the transplant site facilitated rapid fish colonization and diversification, analogous to findings by Hardison et al. (2023) and Sievers et al. (2025), which expounded that restored estuarine *Z. marina* sites provided high-quality habitat for species utilization within 1 year. The increased diversity at the transplant site may have been influenced by the colonizer effect (Sogard, 1989), with resource competition expected to reduce species richness over time (Meakin & Qin, 2020); though notably, no decline in assemblage composition was observed at the transplant site relative to reference sites. Yet the decadal-scale sustainability of fisheries benefits remains uncertain due to the limited 2-year post-restoration monitoring and high documented spatiotemporal fish assemblage fluctuations (Obaza et al., 2022; Tanner et al., 2019). Indeed, the relative isolation of the transplant site (Fig. 1) may have facilitated functional recovery, consistent with evidence that fish utilization is higher in *Z. marina* beds farther from reef habitats (Obaza et al., 2022), likely due to reduced emigration and predation. This may reflect a more resident fish community in perennial *Z. marina* beds on the island, unlike annual beds in other regions requiring seasonal recolonization (Gräfnings et al., 2024).

Quantifying biodiversity is key to understanding ecosystem function (Unsworth et al., 2022). The Button Shell transplant site illustrated no time lag in diversity, surpassing reference site averages within 1 year post-transplant. This finding conforms to previous functional recovery in seagrass restoration studies from other regions (Gagnon et al., 2023; Hardison et al., 2023; Rezek et al., 2019; Ruesink et al., 2019; Titioatchasai et al., 2023) and in a California estuarine system (Beheshti et al., 2022). Indicator species analysis confers similarities in fish assemblages between transplant and reference sites, with species identified by Obaza et al. (2022) occurring equally in both. These results are particularly encouraging, considering many restoration initiatives struggle to reestablish functional community diversity to pre-disturbance or reference site values (Benayas et al., 2009). Seagrasses, like other fast-growing foundational species capable of rapid functional recovery (Grime et al., 2023; Layton et al., 2020; Miller et al., 2024), are an ideal focal species for restoration. Contextualized in polarity to slow-growing foundational species such as temperate reforestation (Case et al., 2023) or coral reefs (Hein et al., 2021), which are undoubtedly critical biodiversity hotspots but require orders of magnitude longer timelines for functional recovery. Noting, however, that not all seagrass ecosystem functions and services return on the same time horizon, with structural and fish functions returning within a year (as we have shown in the present study), whereas recovery of functional carbon sequestration to pre-disturbance levels is implausible (500 to 1000 years) (Ward & Beheshti, 2023; Ward et al., 2021).

Sampling multiple reference sites offers clear quantitative value (Pickett & Parker, 1994), enabling comparisons across broader ecological baselines rather than site-specific disturbances (Underwood, 1992). High interannual variability in fish communities across individual reference sites further evidences the utility of this approach as an avenue to buffer against natural fluctuations (Gagnon et al., 2023). Although definitive causative factors for site differences in fish assemblages (e.g., seagrass bed size, proximity to rocky reef, location of Marine Protected Area) are beyond the scope of this analysis (but see Obaza et al., 2022 and Waters et al., 2023), variation in *Z. marina* health and extent, although less impacted than mainland estuarine systems, may be influenced by stochastic water quality events or anthropogenic disturbances, particularly boat anchoring (Broad et al., 2020; Kelly et al., 2019; Seto et al., 2024). It remains nigh impossible to track every headwind to eelgrass growth, but monitoring numerous reference beds may overcome isolated site-specific impacts (Montefalcone et al., 2008; York et al., 2015) and enhance detection of restoration outcomes (Ward & Beheshti, 2023). While a statistical quantification of these negative impacts is absent from our study, divers observed anchor scars and fragmentation at Ripper’s Cove, which coincided with reduced eelgrass extent and fish diversity—impacts likely linked to high boating and tourism activity (Catalina Chamber of Commerce, 2025; Tompkins & Steller, 2016). The current policy landscape fails to address anchoring restrictions in open-coast seagrass beds, creating substantial data gaps on habitat impacts, feasible risk mitigation strategies, enforcement mechanisms, and economic implications. This regulatory patchiness, combined with the anchoring impacts observed in this study, underscores the urgent need to integrate open-coast seagrass protections into marine spatial planning (Obaza et al., 2022). Ecological moorings (Luff et al., 2019) and boater education are essential for harmonizing policy regimes and conserving this priority habitat.

Fish populations exhibit spatial and temporal variability (Desmond et al., 2002; Holbrook et al., 1994; Maes et al., 2004), making it possible that post-transplant increases in the fish community at Button Shell were obscured by spatiotemporal fluctuations (Hardison et al., 2023). High-resolution Time Lapse Camera (TLC) data complemented diver-based surveys (Bilodeau et al., 2022; Obaza et al., in review), capturing novel species interactions (Fig. 4D), providing insights into fish utilization of open-coast *Zostera* spp. habitats, and bolstering opportunities for education, public engagement, and scientific discovery (Hoeberechts et al., 2015). Although alpha diversity metrics from TLCs showed no statistical seasonal or monthly differences, the data affirm the successful functional development of the transplant site. Despite the expected transplant site progression (i.e., fewer fish in non-vegetative areas compared to mature seagrass; Duffy, 2006), a seasonal gradient was evident (Fig. 7). Consistent patterns were reported in open-coast *Zostera* spp. fish assemblages in the SCB (Waters et al., 2023), while Tanner et al. (2019) expounded direct concurrence with our findings, noting highest fish abundance in summer and lowest in winter at Big Fisherman’s Cove on Catalina Island (site six in our study, Fig. 1). These TLC results lack a balanced sampling design due to missing full seasonal data (i.e., no summer 2024, nor autumn data). That said, the benefits are twofold: (1) the in situ camera data advanced the understanding of species utilization at the transplant site, while demonstrating the efficacy of TLC use in open-coast seagrass systems, and (2) it provided researchers with enhanced opportunities to develop and strengthen outreach capabilities (e.g., time-lapse videos of restoration site expansion). The findings warrant further robust TLC-based investigation (see Bilodeau et al., 2022) to better understand temporal shifts (seasonal and diel) in fish communities and community-wide connectivity across adjacent ecotones.

Examining days when diver-based visual fish surveys overlapped with TLC deployment highlights the value of complementary methodological approaches. On two summer days (July 15, 2022, and July 25, 2023), the TLC recorded three fish from two species and nine fish from three species, while diver-based surveys observed 174 fish from eight species and 326 fish from nine species. Though interestingly, diver-based surveys did not detect the presence of the ESA-listed *Chelonia mydas* (Green Sea Turtle) across all surveys in this study (nor in Obaza et al., 2022), possibly due to the diver effect (Dearden et al., 2010; Dickens et al., 2011), while the TLC captured three separate occurrences. This demonstrates the utility of implementing numerous survey methods in tandem. Waters et al. (2023) affirmed this principle by incorporating SCUBA-based visual surveys and environmental DNA (eDNA) to study fish dynamics in open-coast *Zostera* spp. habitats in the SCB, though the lack of overlapping sampling days effectively reduced direct comparative power given the high spatial and temporal variability in fish populations (Lamy et al., 2018; Obaza et al., 2015). The benefits are further evidenced by scientific monitoring programs that leverage citizen scientists SCUBA divers (Obaza et al., 2024) and recreational fishers (Andrews et al., 2019) to fill data gaps that remain unresolved by conventional methods. Additional complementary tools, like baited remote underwater video (BRUV) systems (Langlois et al., 2010) and diver-operated stereo-video (Harvey et al., 2004), can further enhance data collection. Nonetheless, multiple diverse survey methodologies, taken in amalgam, may reduce individual limitations and provide a more comprehensive view of fish communities in complex systems (French et al., 2021), generating actionable information for resource managers and conservation efforts (Edgar et al., 2016).

## Conclusions

As the region approaches saturation of suitable habitat for seagrass restoration and mitigation areas within bays, harbors, and estuaries caused by climate change and anthropogenic impacts, a pressing challenge emerges in the allocation of adequate space for future projects. The open-coast presents far more unbounded space and greater climate resiliency opportunities than sheltered estuarine systems (Harley et al., 2006; Tan et al., 2020). Yet the dearth of open-coast species-specific and habitat-specific data limits progress (Sanders et al., 2024). Therefore, this project’s successful expansion of seagrass restoration to the open-coast is especially pertinent and may be used as a model for future ecological and managerial regimes.

Given the rapid expansion in area, growth of seagrass structural metrics, and enhancement in the richness and diversity of fish assemblages associated with this study, merged with a current scarcity of open-coast seagrass research, we strongly advocate for additional research aimed at further teasing apart open-coast seagrass mechanistic drivers, seasonal and diel fish assemblage dynamics, and avenues to conduct open-coast restoration at scale. Of note, a recent swell of successful seed-based methodological seagrass restoration has occurred (Govers et al., 2022; Gräfnings et al., 2023; Orth et al., 2020; Unsworth et al., 2019a), and the semi-protected hydrodynamical nature of the leeward side of Catalina Island may present an optimal locale in California to expound open-coast *Zostera* spp. seed dynamics and transplanting approaches. In fact, the Channel Islands archipelago may act as a refuge from the amplification of anthropogenic climate change and the acute and press stressors, even as these same factors catalyze deleterious influence on adjacent mainland nearshore temperate ecotones in the Southern California Bight. And further, it is possible that the extant reference beds on Catalina Island form a metapopulation, and thus, the establishment of a transplant regime that rapidly mirrors natural extant reference beds on the island may allow for further expansion and decrease the risk of *Zostera* spp. extirpation by inhabiting distinct areas with fine-scale local adaptations (DuBois et al., 2022).

This study illuminates the tangibility of successful marine restoration and provides avenues to contribute to multi-dimensional benefits from fisheries enhancement to biodiversity conservation and supporting critical habitat for endangered species. The further disentanglement of the realized niche occupied by *Zostera* spp. in open-coast environments will aid in the development of a holistic comprehension designed at guiding an effective, scalable, and ecologically significant restoration regime in the Southern California Bight.

## Supplementary Information

Below is the link to the electronic supplementary material.

ESM 1 (DOCX 11.5 MB)

## Data Availability

The dataset generated during the current study will be made available from the corresponding author on reasonable request.
