# Supplementary material for: Open-Coast Eelgrass (Zostera marina) Transplant Catalyzes Rapid Mirroring of Structure and Function of Extant Eelgrasses
Source: Estuaries Coast. 2025 Sep 30;49(1):2. doi: 10.1007/s12237-025-01609-x (PMC12484352; doi:10.1007/s12237-025-01609-x)
Supplement: Supplementary file 1 — (DOCX 11.5 MB) [file 12237_2025_1609_MOESM1_ESM.docx]

**Open-coast eelgrass (*Zostera marina)* transplant catalyzes rapid mirroring of structure and function of extant eelgrasses**

**Electronic Supplementary Materials**

**Journal:** Estuaries and Coasts

**Authors:**

Rilee D. Sanders^1,2^*, Adam K. Obaza^2^, David W. Ginsburg^3^, Olivia C. Carmack^2^, Benjamin C. Grime^4^, Heather Burdick^5^, Tom K. Ford^5^ and James J. Leichter^1^

**Affiliations:**

^1^Scripps Institution of Oceanography, University of California San Diego, La Jolla, CA, USA.

^2^Paua Marine Research Group, Long Beach, CA, USA.

^3^Environmental Studies Program, University of Southern California, Los Angeles, CA, USA.

^4^The Nature Conservancy, Los Angeles, CA, USA.

^5^The Bay Foundation, Los Angeles, CA, USA.

***Corresponding author:**

rdsanders@ucsd.edu


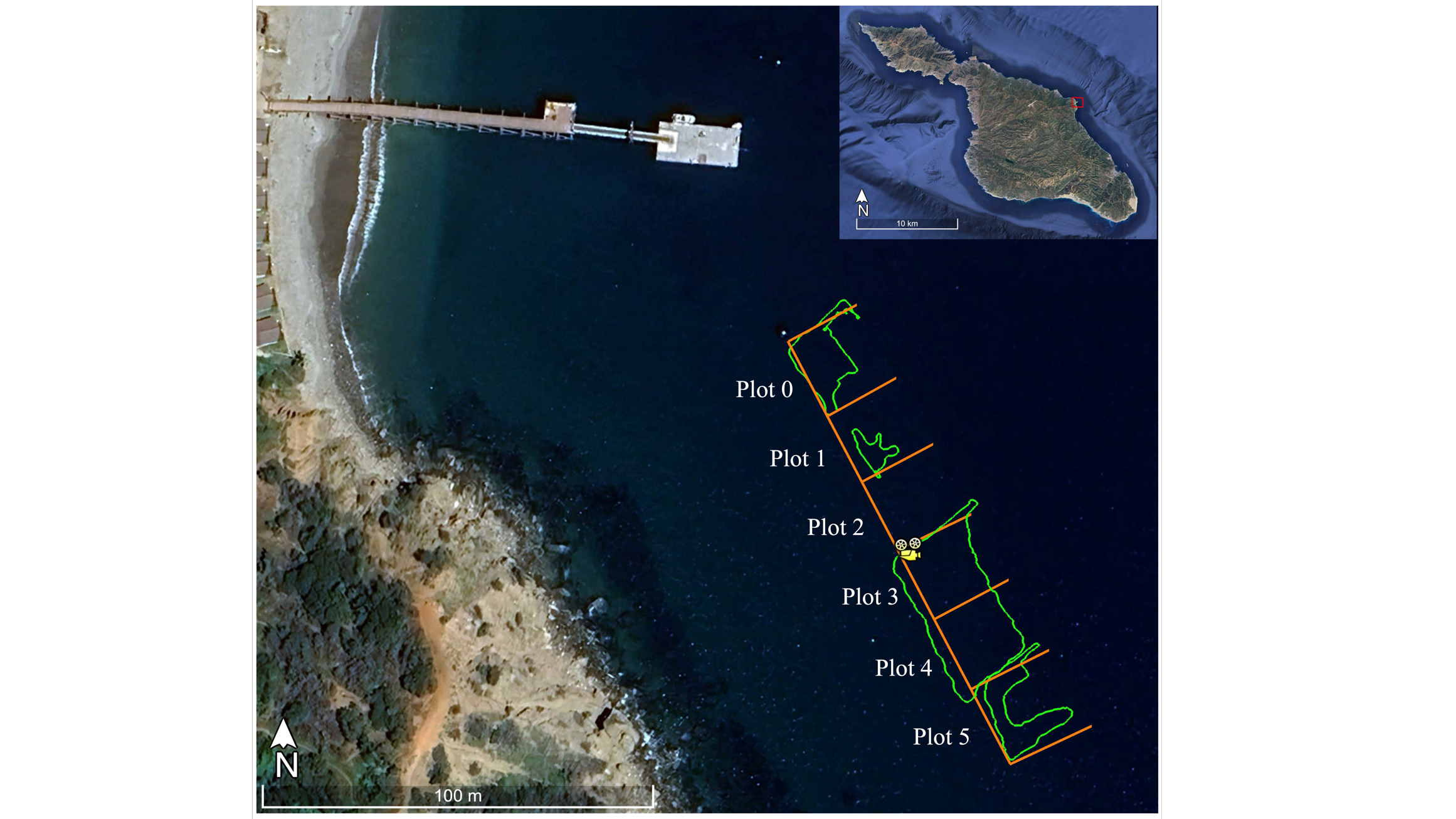


**Fig S1** *Zostera marina* (common eelgrass) transplanting design at the Button Shell transplant site, Catalina Island. The site was delineated utilizing a 120 m baseline with seven 21 m perpendicular transects (orange lines), creating six replicate plots (420 m^2^ each) along the same depth strata gradient. Transplant plots zero, one, and five received the single shoot method, transplant plots three and four received the bundle shoot method, and transplant plot two was left fallow. Green polygons represent the perimeter of transplanted *Z. marina* at the completion of transplanting activities in June 2022. The locations of the time lapse camera (TLC) and biophysical sensor array are denoted on the map with the camera logo.
